# Effect of lifestyle modification and atorvastatin on dyslipidemia and endothelial dysfunction markers in children with steroid resistant nephrotic syndrome

**DOI:** 10.1590/2175-8239-JBN-2025-0212en

**Published:** 2026-04-17

**Authors:** Vanshika Kakkar, Abhijeet Saha, Prajal Agrawal, Sunita Sharma, Anita Nagia, Prachi Singh, Harish K. Pemde

**Affiliations:** 1 Lady Hardinge Medical College, Department of Paediatrics, Division of Pediatric Nephrology, Kalawati Saran Children Hospital, New Delhi, India.; 2 Lady Hardinge Medical College, Department of Pathology, Sucheta Kriplani Hospital, New Delhi, India.

**Keywords:** Dyslipidemias, Endothelial dysfunction, Lifestyle modifications, Nephrotic Syndrome, Atorvastatin

## Abstract

**Introduction:**

Persistent dyslipidemia in children with steroid resistant nephrotic syndrome (SRNS) causes endothelial dysfunction resulting in adverse cardiovascular outcomes.

**Methods:**

Thirty-four patients aged 4–18 years newly diagnosed with SRNS and no previous statin use were enrolled in this longitudinal observational study. Serum levels of total cholesterol, LDL cholesterol, and endothelial dysfunction markers (PSCK-9, E-selectin) were measured at baseline and after 12 weeks of lifestyle modifications in all patients, and atorvastatin was prescribed to those with LDL-cholesterol > 160 mg/dL. Primary outcome measure was change in plasma levels of total cholesterol, LDL cholesterol, PSCK-9, and E-selectin at 12 weeks. The secondary outcome was the correlation of PSCK-9 and E-selectin with lipid profile, blood pressure, BMI, serum albumin, and urine-protein creatinine ratio.

**Results:**

There was a significant decline in total cholesterol, LDL cholesterol, E-selectin, and PCSK-9 levels at 12 weeks of lifestyle modification and atorvastatin therapy. In a subgroup analysis between the statin group (n = 16) and the non-statin group (n = 18), both groups showed a significant decline in lipid profile parameters with a more pronounced decline in the statin subgroup. Endothelial dysfunction markers (E-selectin and PSCK-9) showed a declining trend in both subgroups, but the decline was not statistically significant in the statin group.

**Conclusion:**

Dietary and lifestyle modifications led to a decline in endothelial dysfunction markers and an improvement in lipid profile. Statins helped improve dyslipidemia but did not significantly improve endothelial dysfunction markers at 12 weeks.

## Introduction

Nephrotic syndrome is a glomerular disease that is characterized by heavy proteinuria, hypoalbuminemia, and edema^1^. The incidence varies from 1.15–16.9 /100,000 children per year^2^. About 85% of children achieve remission following standard therapy with prednisolone, but 10–15% of patients fail to achieve complete remission and are termed as steroid resistant nephrotic syndrome (SRNS)^3^. Serum cholesterol, triglycerides, and lipoproteins are raised in children with nephrotic syndrome and persist in those with SRNS^3^. Persistent hyperlipidemia not only injures glomeruli and tubules but also results in endothelial damage, which is an early marker for atherosclerosis^4,5^. Endothelial dysfunction tends to be more pronounced in children with frequently relapsing nephrotic syndrome (FRNS) and SRNS resulting in accelerated atherosclerosis, ischemic heart disease, stroke, thrombosis, and progressive kidney disease^4,6,7,8,9,10^. Hypertension, hypoalbuminemia, and hypercoagulable state also contribute to cardiovascular morbidity in these children^3^. Studies have shown that serum levels of E-selectin, proprotein convertase subtilisin/kexin type 9 (PCSK9), soluble thrombomodulin (sTM), von-willebrand factor (vWF), tissue plasminogen activator (tPA), plasminogen activator inhibitor-1 (PAI-1), and soluble vascular cell adhesion molecule-1 (VCAM-1) are altered as a result of damage to endothelial cells, which may serve as plasma markers of endothelial dysfunction^11,12,13^. National and international guidelines have proposed lifestyle modification and use of statins for the treatment of dyslipidemia in children with nephrotic syndrome to prevent cardiovascular morbidity and chronic kidney disease in this population^14,15^. The aim of this study was to evaluate the effect of lifestyle modifications and statins on lipid profile (low-density lipoprotein cholesterol and total cholesterol) and on markers of endothelial dysfunction (PCSK9 and E-Selectin) in patients with SRNS.

## Methods

This longitudinal observational study was conducted at a tertiary care teaching center from November 2019 to October 2021 after approval from the institutional ethical committee (IEC). Children aged 4–18 years with a diagnosis of SRNS according to guidelines of Indian society of pediatric nephrology were included while patients with SRNS receiving pharmacologic therapy (statins) for hyperlipidemia at the time of enrolment were excluded from the study^3^. Demographic and clinical details including anthropometry, blood pressure, and features of steroid toxicity were recorded. Blood samples were collected for complete blood count, serum electrolytes, urea, creatinine, total protein, albumin, total cholesterol, low-density lipoprotein cholesterol (LDL-C), triglycerides, high-density lipoprotein cholesterol (HDL-C), non-HDL-C, and markers of endothelial dysfunction (PCSK9, sE-selectin) and urine samples were collected for spot urine protein creatinine ratio of patients enrolled in the study. These samples were collected at the time of enrollment and after 12 weeks of standard treatment. Plasma was obtained by blood sample centrifugation at 2800 *g* for 10 minutes for measurement of endothelial markers. The sample was stored at -20°C until analysis. Quantitative assessment of endothelial markers was performed with ELISA technique using specialized kits for each marker (Human PCSK9 kit manufactured by Biovendor-Laboratorni Medicine A.S. Czech Republic and sE-Selectin kit manufactured by Shanghai Coon Koon Biotech Co. Ltd). The measurement was performed on an Erba Lisa Scan EM ELISA machine.

Children with SRNS received standard therapy with calcineurin inhibitors (tacrolimus or cyclosporine) and tapering dose of prednisolone^3^. All patients received lifestyle modifications, which included dietary restrictions and a standard regimen of physical exercise for the management of dyslipidemia. Dietary advice included reducing the intake of saturated fats and cholesterol according to CHILD-1 guidelines, which restricts saturated fat to < 10% of daily calories and cholesterol to 300 mg/day. These restrictions are further tightened in the CHILD-2 diet with saturated fat < 7% and cholesterol < 200 mg/day^16^. Leisure screen time was limited to less than 2 hours per day, and moderate physical activity for 1-hr/day with vigorous physical activity at least 3 days a week were also advised^16^. Diet was recorded twice a week using the estimated record method of dietary assessment in structured diet charts provided to the patients. Dietary intake was evaluated in each follow up visit to ensure compliance. Pharmacologic therapy with atorvastatin was prescribed in addition to lifestyle modifications to children with a serum LDL cholesterol > 160 mg/dl at the time of enrolment^15,16,17^. Atorvastatin was given to children aged 4 years and above at a fixed dose of 10 mg/day^11,15^. Height, weight, serum CPK-MB, serum SGOT, and SGPT were monitored every 4 weeks in patients receiving atorvastatin. All patients received therapy with enalapril (0.2-0.3 mg/kg) for its anti-proteinuric effect. Antihypertensives were prescribed for the management of hypertension^3^.

The primary outcome measure was change in plasma levels of total cholesterol, LDL-C, E-selectin and PCSK9 in children with SRNS after 12 weeks of standard treatment. The secondary outcome measure was correlation of plasma levels of E-selectin and PCSK9 with total cholesterol, LDL-C, non HDL-C , BMI, BP, eGFR, spot urine protein creatinine ratio, and serum albumin at diagnosis of SRNS and after 12 weeks of standard treatment.

Sample size was calculated using Epi Info software. Among patients with nephrotic syndrome, mean plasma PCSK level was 15.13 with a standard deviation of 4.99, which was significantly higher than that of age- and gender-matched controls (p < 0.001) in a previous study by Jin et al.^18^. Considering a mean difference of 5.67, as reported in this study, a sample size of 12 patients was obtained for PCSK9 at 80% power and 95% confidence interval. Using reference values for mean and SD for E-selectin from a previous study conducted at our institute, the sample size was estimated to be 10. We recruited a larger sample of 34 patients with SRNS in this study.

### Statistical Analysis

Data was analyzed using IBM SPSS Statistics (version 23.0). Statistical significance was considered at p < 0.05. Categorical data was presented as frequencies and percentages. Continuous data was presented as mean and standard deviation for normally distributed data or median and interquartile range for non-normal distribution. Kolmogorov-Smirnov test was used to check the normality of the quantitative data. The paired sample t-test was used for normally distributed data and the paired Wilcoxon test was used for skewed data to compare patients at the two time points. Pearson’s correlation for (for normally distributed data) and Spearman’s correlation (for non-normally distributed data) were used to explore the linear correlation between two continuous variables. Generalized estimating equations were used for comparison between two groups over time.

## Results

The study group included 34 patients with a diagnosis of SRNS who were prospectively followed up for 12 weeks. Mean age at nephrotic syndrome onset was 63 months and mean age at the time of diagnosis of SRNS was 88 months. Deranged lipid profile was found in 24 (71%) patients at SRNS diagnosis. At diagnosis, 14 (41%) patients were hypertensive, with 13 (38%) patients having stage I hypertension and 1 (3%) patient having stage II hypertension. There was a significant improvement in BP percentiles over 12 weeks. BP normalized in 12 patients with stage I hypertension and in the one patient with stage II hypertension (p = 0.004) following 12 weeks of intervention. We also compared the change in E-selectin and PCSK9 in the BP percentile subgroups 50–90%, 90–95%, 95–95+12% and > 95+12% using Kruskal Wallis test and found no significant difference (p = 0.239 and p = 0.913) in the 4 subgroups. On evaluating dietary intake over the 12-week study period, the average daily fat intake was 24.93 g/day, accounting for 18.9% of total caloric intake. The average daily saturated fat intake was 7.6 g/day, accounting for 6.3% of total caloric intake. The average daily cholesterol intake was 55.42 mg, all of which were well within the advised range (Table 1). There was a marked improvement in anthropometric parameters (Table 2). Spot urine protein-creatinine ratio showed a significant decline (p < 0.001) and an improvement in serum albumin levels was seen (p = 0.002), as shown in Table 2. Complete remission was achieved in 30% of patients and partial remission was achieved in 56% patients. A significant decline was found in mean levels of total cholesterol, LDL-C, triglycerides, and non-HDL-C at 12 weeks of dietary and lifestyle modifications and atorvastatin therapy (Table 3). Both PCSK9 and E-selectin demonstrated a significant reduction (p < 0.008 and p < 0.029 for PCSK-9 and E-selectin, respectively). Children who received atorvastatin in addition to lifestyle modifications showed a greater reduction in serum cholesterol, LDL cholesterol, triglyceride, and non-HDL cholesterol as compared to children receiving only lifestyle modification. The serum levels of endothelial markers (PCSK9 and E-selectin) showed a declining trend (not statistically significant) in the group receiving statin therapy (Table 4).

**Table 1:** Dietary intake over 12 weeks of lifestyle modifications

Diet	Mean (SD)	Median (IQR)
Average Daily Caloric Intake (Kcal)	1193.85 ± 323.42	1181.00 (1000.00–1320.00)
Average Daily Protein Intake (g)	43.41 ± 10.33	41.00 (39.00–47.50)
Caloric Deficit (%)	31.07 ± 14.04	27.00 (21.00–43.00)
Protein Deficit (%)	8.22 ± 12.04	0.00 (0.00–12.50)
Average Daily Fat Intake (g)	24.93 ± 9.59	23.00 (16.50–33.00)
Calories from Fat (%)	18.90 ± 6.15	17.00 (14.50–24.35)
Average Daily Saturated Fat Intake (g)	7.60 (5.50–10.50)	7.60 (5.50–10.50)
Calories From Saturated Fat (%)	6.30 ± 3.05	6.00 (4.05–7.50)
Average Daily Cholesterol Intake (mg)	55.42 ± 50.92	49.00 (20.00–60.00)

**Table 2:** Anthropometric and laboratory parameters at diagnosis and at 12 weeks

S. No.	Parameter	SRNS cases at diagnosis (n = 34)	SRNS cases at 12 weeks (n = 34)	p-value
1.	Weight (kg)	23.35 ± 8.25 22.10 (16.60–26.60)	22.01 ± 8.25 20.30 (16.00–25.10)	0.003^1^
2.	Height (cm)	115.32 ± 18.63 113.90 (100.50–131.20)	117.53 ± 18.62 115.00 (102.00–132.80)	<0.001^2^
3.	BMI (kg/m^2^)	17.10 ± 2.04 16.90 (15.70–18.00)	15.39 ± 1.74 15.50 (14.00–16.40)	<0.001^1^
4.	Urea (mg/dL)	35.41 (26.16) 30.00 (21.18–44.28)	25.60 (19.35) 21.50 (17–26)	<0.010^1^
5.	Creatinine (mg/dL)	0.36 (0.19) 0.30 (0.21–0.45)	0.34 (0.11) 0.30 (0.28–0.40)	<0.820^1^
6.	eGFR (mL/min/1.75 m^2^)	154.71 (58.34) 146.00 (123–196)	151.91 (45.19) 144.00 (127–169)	<0.775^1^
7.	Total Protein (g/dL)	5.47 (1.26) 5.60 (4.26–6.47)	6.47 (1.08) 6.80 (6.40–7.20)	<0.001^1^
8.	Albumin (g/dL)	3.01 (1.06) 3.10 (2.10–3.88)	3.67 (1.03) 4.00 (3.80–4.26)	<0.002^1^
9.	Up:Uc	4.77 (6.47) 2.42 (1.72–7.22)	1.17 (2.06) 0.48 (0.19–0.98)	<0.001^1^

Notes – Values are mean ± SD and median ± IQR. Significant at p < 0.05.

^1^Paired Wilcoxon test;

^2^paired t-test.

**Table 3:** Lipid profile, e-selectin and pcsk 9 levels at diagnosis and at 12 weeks

S. No.	Parameter	SRNS cases at diagnosis	SRNS cases at 12 weeks	p-value
1.	Total cholesterol (mg/dL)	273.71 ± 124.70 225.50 (188.00–312.00)	177.59 ± 91.66 159.50 (131.25–180.75)	<0.001^1^
2.	LDL (mg/dL)	160.50 ± 91.88 126.50 (95.50–213.00)	100.85 ± 75.74 75.00 (63.50–112.75)	<0.001^1^
3.	Non-HDL cholesterol (mg/dL)	193.00 ± 119.96 145.50 (112.25–246.00)	118.03 ± 88.39 94.00 (77.50–118,75)	<0.001^1^
4.	TG (mg/dL)	234.38 ± 138.13 214.50 (120.00–335.00)	150.60 ± 106.13 127.00 (87.50–172.25)	0.001^1^
5.	HDL (mg/dL)	80.71 ± 22.82 81.00 (61.50–97.25)	59.56 ± 11.82 58.00 (541.50–68.00)	<0.001^2^
6.	E-selectin (pg/ml)	614.24 ± 112.38 631.25 (575.25–668.00)	544.37 ± 137.91 561.50 (427.50–657.75)	0.029^1^
7.	PCSK9 (ng/mL)	518.25 ± 222.34 521.90 (388.25–706.35)	373.25 ± 198.71 326.50 (217.25–504.25)	0.008^1^

Notes – Values are mean ± SD or median ± IQR. *Significant at p < 0.05.

^1^Paired Wilcoxon test;

^2^paired t-test.

**Table 4:** Subgroup analysis of patients with and without statin therapy at baseline and 12 weeks

S. No	Parameter	Statin	Baseline	12 weeks	p value	Overall p value^3^
1.	Cholesterol (mg/dL)	Yes (n = 16)	354.56 (136.61)	201.38 (121.01)	0.002^1^	
		No (n = 18)	201.83 (45.80)	156.44 (48.82)	0.001^1^	0.006
2.	LDL Cholesterol (mg/dL)	Yes (n = 16)	220.12 (99.40)	125.88 (99.66)	0.012^1^	
		No (n = 18)	107.50 (36.73)	78.61 (35.46)	0.001^1^	0.021
3.	Non-HDL Cholesterol (mg/dL)	Yes (n = 16)	267.62 (135.53)	143.69 (117.92)	0.006^1^	
		No (n = 18)	126.67 (42.76)	95.22 (41.54)	0.004^1^	0.019
4.	Triglyceride (mg/dL)	Yes (n = 16)	311.00 (146.99)	199.12 (131.54)	0.013^1^	
		No (n = 18)	166.28 (86.81)	107.39 (49.04)	0.053^1^	0.226
5.	HDL Cholesterol (mg/dL)	Yes (n = 16)	86.94 (25.58)	75.17 (19.10)	<0.001^2^	
		No (n = 18)	57.69 (11.43)	61.22 (12.23)	0.012^2^	0.040
6.	Albumin (g/dL)	Yes (n = 16)	2.69 (1.04)	3.37 (1.24)	0.083^1^	
		No (n = 18)	3.28 (1.03)	3.94 (0.73)	<0.001^1^	0.977
7.	Urine Protein creatinine ratio	Yes (n = 16)	6.72 (8.76)	1.62 (2.71)	<0.001^1^	
		No (n = 18)	3.04 (2.61)	0.76 (1.18)	<0.001^1^	0.089
8.	PCSK9 (ng/mL)	Yes (n = 16)	509.11 (237.74)	425.19 (198.10)	0.252^1^	
		No (n = 18)	526.38 (214.34)	327.08 (192.94)	0.021^1^	0.251
9.	E-selectin (pg/mL)	Yes (n = 16)	583.03 (111.71)	557.34 (130.67)	0.528^1^	
		No (n = 18)	641.97 (108.50)	532.83 (146.81)	0.021^1^	0.151

Notes – Values are mean ± SD. *Significant at p < 0.05.

^1^Paired Wilcoxon test;

^2^paired t-test.

^3^generalized estimating equation.

We compared the percentage change in lipid profile parameters and endothelial dysfunction markers with remission status and found a similar change in total cholesterol (p = 0.180), LDL-C (p = 0.257), triglycerides (p = 0.355), non-HDL-C (p = 0.097), PCSK9 (p = 0.138), and E-selectin (p = 0.902) in the non-remission, partial remission, and complete remission subgroups (Table 5).

**Table 5:** Comparison of lipid profile, e-selectin, pcsk 9 with remission status

S. No.	Parameter (Percentage change)	Remission status			p value
		None (n = 5)	Partial (n = 19)	Complete (n = 10)	
1.	LDL	-25.31 (-31.71– -15.25)	-29.56 (-52.32–8.12)	-43 (-50.82– -32.34)	0.257
2.	Total Cholesterol	-24.17 (-26.12– -13.76)	-27.81 (-48.06– -9.11)	-38.32 (-53.68– -29.73)	0.180
3.	Triglycerides	-20.87 (-44.08– -20.42)	-27.86 (-56.17–13.09)	-56.24 (-75.47– -4.81)	0.355
4.	Non-HDL Cholesterol	-21.31 (-27.41– -11.78)	-22.96 (-50.00.42–6.67)	-46.58 (-59.22– -37.14)	0.097
5.	E-Selectin	5.56 (-31.17–6.49)	-12.09 (-23.46–9.33)	-7.16 (-21.03– -3.78)	0.902
6.	PCSK9	-24.66 (-29.25–28.17)	-29.64 (-47.95–23.46)	-60.27 (-79.36– -21.58)	0.138

Notes – Values are median ± IQR. *Significant at p < 0.05. Kruskal-Wallis test.

PCSK9 showed a significantly positive correlation with urine protein-creatinine ratio (r = 0.36; p = 0.035) and a significantly negative correlation with serum albumin (r = -0.33; p = 0.054) at 12 weeks. E-selectin showed a positive correlation with BMI at diagnosis of SRNS (r = 0.52; p = 0.002). In patients receiving atorvastatin, no significant elevation in serum creatinine kinase and transaminase levels was observed during the 12-week follow up. A significant improvement was noted in weight and height of patients at 12 weeks. No other adverse event was reported in the atorvastatin therapy subgroup.

## Discussion

Endothelial injury in nephrotic syndrome results from a variety of causes, including hypoalbuminemia, dyslipidemia, and oxidative stress^13^. Lipid abnormalities in nephrotic syndrome occur due to both increased lipid biosynthesis and decreased clearance, with decreased clearance being the predominant factor. Plasma levels of total cholesterol, LDL-C, very-low-density lipoprotein cholesterol (VLDL-C), and triglycerides are increased and remain persistently high in children with SRNS^5^. Exposure to atherogenic factors, including increased serum lipids, alters the endothelial phenotype to a non-adaptive state known as endothelial dysfunction resulting in release of various pro-inflammatory and pro-thrombotic molecules from activated endothelial cells including vWF, tPA, VCAM-1, and E-selectin^19^. PCSK 9 is a serine protease that is mainly produced by the liver. It binds to the extracellular domain of the LDL-LDL-R complex on hepatocytes, preventing its recycling. PCSK 9 also interacts with CD36 scavenger receptors present on macrophages for uptake of oxidized LDL and therefore may play a direct role in promoting atherosclerosis^20^.

Subclinical abnormalities in blood vessels and myocardium begin developing during childhood and manifest clinically as myocardial infarction, angina, and cerebrovascular accidents including thromboembolism later in life^4,21^. Our study demonstrated that markers of endothelial dysfunction, PCSK9 and E-selectin, declined significantly after 12 weeks of dietary and lifestyle modification indicating that regulating diet and engaging in adequate physical activity play an important role in reversing endothelial dysfunction in children with nephrotic syndrome. When evaluating diet composition in our patients, we found a significantly higher intake of saturated fat than recommended at baseline. However, with periodic follow-up on compliance to advised modifications and repeated dietary counseling, most patients reduced their dietary intake to recommended levels, Therefore, involvement of a dietician and adherence to the dietary regimen are necessary.

The effects of lifestyle changes on the treatment of dyslipidemia and endothelial dysfunction vary. Woo et al.^22^ studied the effect of a hypocaloric low-fat diet and exercise on flow-mediated vasodilation and reported a decrease in serum cholesterol and improvement in endothelial function in the short term (at 6 weeks), with reversal to near normal levels with sustained intervention for 1 year. Engler et al.^23^ examined the effects of the National Cholesterol Education Program Step II diet on endothelium-dependent vasodilation in children with dyslipidemia. The dietary modifications significantly reduced the LDL-C concentration but failed to improve endothelial function during the 6-month trial. The subgroup analysis of patients with no remission (14%), partial remission (30%), and complete remission (56%) showed no significant difference in change in total cholesterol, LDL cholesterol, triglycerides, and non-HDL cholesterol levels after 12 weeks of standard treatment, suggesting that remission status does not have a significant impact on improvement of endothelial dysfunction.

It is well known that dyslipidemia can alter endothelial phenotype resulting in endothelial dysfunction. In our study, 71% of patients had deranged lipid profile at SRNS onset, with increased total cholesterol in 71% of patients and elevated LDL cholesterol in 47% of patients. Watts et al.^24^ demonstrated that endothelial dysfunction in patients with nephrotic syndrome is similar to those with hyperlipidemia, showing a significantly positive correlation with serum LDL, concluding that dyslipidemia plays a role in endothelial dysfunction. In our study, the mean total cholesterol (177.59 mg/dL) and LDL cholesterol (100.85 mg/dL) after treatment were similar to controls in the study by Watts et al.^19^. Several studies have demonstrated that patients with nephrotic syndrome have higher serum PCSK9 levels than controls, and that there is a positive correlation with total cholesterol and HDL cholesterol at baseline, indicating that PCSK9 plays a significant role in contributing to dyslipidemia^18,25^. A positive correlation was also found between serum PCSK9 and urine protein-creatinine ratio and a negative correlation was observed with serum albumin following treatment. Doiron et al.^26^ found a similar correlation between proteinuria and PCSK9 levels in patients with nephrotic and non-nephrotic range proteinuria, as well as in controls. The present study did not find a significant correlation between endothelial markers and total cholesterol and LDL cholesterol.

Children with nephrotic syndrome also experience frequent infections, oxidative stress, hypertension, and drug toxicity of both steroidal and steroid-sparing immunosuppressants such as calcineurin inhibitors, all of which increase the risk of endothelial dysfunction and cardiovascular events^27,28^. In our study, 41% of patients had hypertension at SRNS diagnosis, with a significant improvement in BP percentile over 12 weeks. Hypertension, by various mechanisms, is known to cause endothelial dysfunction^29^. We did not find a statistically significant difference in the change in E-selectin and PCSK9 levels between patients with and without hypertension.

We prescribed tacrolimus to all SRNS patients in our study. Pełkowska et al.^30^ compared endothelial dysfunction in SRNS patients in remission on cyclosporine therapy and nephrotic patients with relapse, and reported similar endothelial dysfunction, indicating that CNI plays a role in endothelial dysfunction. Studies in post-renal transplant patients evaluating the effect of cyclosporine and tacrolimus on endothelial dysfunction have reported tacrolimus to cause less endothelial dysfunction than cyclosporine^31,32^. All patients in our study received ACE inhibitors for its antiproteinuric effect. ACE inhibitors, by increasing availability of nitric oxide and by reducing oxidative stress and inflammation mediated by oxidized LDL, play a role in improving endothelial dysfunction^29^. We did not perform genetic analysis in our patients; therefore, we could not evaluate if genetic or immunological origin of the disease has an impact on children with SRNS. Kaukinen et al.^33^ evaluated the glomerular endothelium in children with congenital nephrotic syndrome (NPHS-1 mutation) and demonstrated that the expression of adhesion molecules like intercellular adhesion molecule 1 (ICAM-1) was increased in NPHS1-positive kidney glomeruli as compared to controls. In children with FSGS, circulating endothelial cells and transforming growth factor beta are significantly elevated^34^.

A subgroup analysis was done among children who received atorvastatin and those who did not receive it, in addition to the dietary management that was advised to all children as part of standard treatment for dyslipidemia. Atorvastatin was given at a fixed dose of 10 mg. No clinical or biochemical side effects were observed in any patient on atorvastatin therapy. Various studies have demonstrated the safety of statins in pediatric patients with hypercholesterolemia^35,36^. The trend of change in markers of endothelial dysfunction, lipid profile, urine protein-creatinine ratio, and albumin over 12 weeks of therapy was compared between the two groups. We found a significant decline in total cholesterol, LDL cholesterol, and non-HDL cholesterol in patients both with or without statins. However, there was a significant difference in the trend of decline in these parameters between the two groups, implying that statins do have a role in treating dyslipidemia. There was a significant decrease in proteinuria (spot urine protein creatinine ratio) in both statin and non-statin subgroups; however, the decline in proteinuria was similar in both subgroups. Coleman and Watson^37^ reported a significant and sustained effect of simvastatin, while dietary intervention had only a modest effect on the lipid profile. The endothelial dysfunction markers showed a declining trend, but this decline did not reach statistical significance in the group of patients receiving statins over 12 weeks of therapy, suggesting that endothelial dysfunction persists despite statin therapy, at least in the short term in SRNS patients. Whether extending statin therapy beyond 12 weeks in SRNS patients decreases endothelial dysfunction needs to be examined.

## Conclusion

Dietary and lifestyle modifications led to a decline in endothelial dysfunction markers and an improvement in lipid profile parameters in patients with SRNS. Lifestyle modifications, which include dietary restrictions and increased physical activity, are the first step for preventing and treating dyslipidemia and other cardiovascular disease risk factors. However, adherence to lifestyle changes is a challenge and requires periodic monitoring. The use of statin therapy helped improve the lipid profile, but statins did not significantly decrease the levels of endothelial dysfunction markers in the short term. Our study was limited by a short follow-up period. Long term prospective studies on the effects of statins on endothelial dysfunction and the use of novel therapies like PCSK9 inhibitors are needed, as these drugs may serve as important targets for alleviating endothelial dysfunction in proteinuric children.

## Data Availability

The datasets generated and analyzed during the current study are available from the corresponding author on request.
