# Supplementary material for: Effect of lifestyle modification and atorvastatin on dyslipidemia and endothelial dysfunction markers in children with steroid resistant nephrotic syndrome
Source: J Bras Nefrol. 2026 Apr 17;48(2):e20250212. doi: 10.1590/2175-8239-JBN-2025-0212en (PMC13132119; doi:10.1590/2175-8239-JBN-2025-0212en)
Supplement: Translation

# Translation: Efeito da modificação do estilo de vida e da atorvastatina na dislipidemia e nos marcadores de disfunção endotelial em crianças com síndrome nefrótica resistente a esteroides

**DOI:** 10.1590/2175-8239-JBN-2025-0212pt

**Authors:** Vanshika Kakkar, Abhijeet Saha, Prajal Agrawal, Sunita Sharma, Anita Nagia, Prachi Singh, Harish K. Pemde

**Affiliations:** 1 Lady Hardinge Medical College, Department of Paediatrics, Division of Pediatric Nephrology, Kalawati Saran Children Hospital, New Delhi, Índia.; 2 Lady Hardinge Medical College, Department of Pathology, Sucheta Kriplani Hospital, New Delhi, Índia.

**Keywords:** Dislipidemias, Disfunção endotelial, Modificações no estilo de vida, Síndrome Nefrótica, Atorvastatina

## Abstract

**Introdução:**

Dislipidemia persistente em crianças com síndrome nefrótica resistente a esteroides (SNRE) causa disfunção endotelial, resultando em desfechos cardiovasculares adversos.

**Métodos:**

Trinta e quatro pacientes, entre 4 e 18 anos, recém-diagnosticados com SNRE e sem uso prévio de estatinas, foram incluídos neste estudo observacional longitudinal. Níveis séricos de colesterol total, colesterol LDL e marcadores de disfunção endotelial (PCSK9, E-selectina) foram mensurados no início e após 12 semanas de modificações no estilo de vida em todos os pacientes; a atorvastatina foi prescrita àqueles com colesterol LDL > 160 mg/dL. O desfecho primário foi a alteração nos níveis plasmáticos de colesterol total, colesterol LDL, PCSK9 e E-selectina após 12 semanas. O desfecho secundário foi a correlação de PCSK9 e E-selectina com perfil lipídico, pressão arterial, IMC, albumina sérica e relação proteína/creatinina urinária.

**Resultados:**

Observou-se redução significativa nos níveis de colesterol total, colesterol LDL, E-selectina e PCSK9 após 12 semanas de modificação do estilo de vida e terapia com atorvastatina. Na análise de subgrupos entre grupo estatina (n = 16) e grupo sem estatina (n = 18), ambos apresentaram redução significativa nos parâmetros do perfil lipídico, com redução mais acentuada no subgrupo estatina. Os marcadores de disfunção endotelial (E-selectina, PCSK9) apresentaram tendência de redução em ambos os subgrupos; porém, no grupo estatina, a redução não foi estatisticamente significativa.

**Conclusão:**

Modificações dietéticas e no estilo de vida promoveram redução dos marcadores de disfunção endotelial e melhora no perfil lipídico. As estatinas contribuíram para melhora da dislipidemia, mas não melhoraram significativamente os marcadores de disfunção endotelial em 12 semanas.

## Introdução

A síndrome nefrótica é uma doença glomerular caracterizada por proteinúria maciça, hipoalbuminemia e edema^1^. Sua incidência varia de 1,15 a 16,9/100.000 crianças por ano^2^. Aproximadamente 85% das crianças alcançam a remissão após terapia padrão com prednisolona; no entanto, 10–15% dos pacientes não atingem a remissão completa e são classificados como síndrome nefrótica resistente a esteroides (SNRE)^3^. Os níveis séricos de colesterol, triglicerídeos e lipoproteínas encontram-se elevados em crianças com síndrome nefrótica e persistem naquelas com SNRE^3^. A hiperlipidemia persistente não apenas lesiona os glomérulos e os túbulos, como também resulta em dano endotelial, considerado um marcador precoce de aterosclerose^4,5^. A disfunção endotelial tende a ser mais pronunciada em crianças com síndrome nefrótica com recidivas frequentes (SNRF) e SNRE, levando à aterosclerose acelerada, doença cardíaca isquêmica, acidente vascular cerebral, trombose e doença renal progressiva^4,6,7,8,9,10^. Hipertensão, hipoalbuminemia e estado de hipercoagulabilidade também contribuem para a morbidade cardiovascular nessas crianças^3^. Estudos demonstraram que os níveis séricos de E-selectina, proproteína convertase subtilisina/kexina tipo 9 (PCSK9), trombomodulina solúvel (sTM), fator de *von Willebrand* (vWF), ativador tecidual do plasminogênio (tPA), inibidor do ativador do plasminogênio-1 (PAI-1) e molécula de adesão celular vascular solúvel-1 (VCAM-1) encontram-se alterados em decorrência de danos nas células endoteliais, podendo atuar como marcadores plasmáticos de disfunção endotelial^11,12,13^. Diretrizes nacionais e internacionais propõem a modificação do estilo de vida e o uso de estatinas para o tratamento da dislipidemia em crianças com síndrome nefrótica, com o objetivo de prevenir a morbidade cardiovascular e a doença renal crônica nessa população^14,15^. O objetivo deste estudo foi avaliar o efeito das modificações no estilo de vida e das estatinas no perfil lipídico (colesterol de lipoproteína de baixa densidade e colesterol total) e nos marcadores de disfunção endotelial (PCSK9 e E-selectina) em pacientes com SNRE.

## Métodos

Este estudo observacional longitudinal foi realizado em um centro de ensino de cuidados terciários, entre novembro de 2019 e outubro de 2021, após aprovação do Comitê de Ética Institucional (CEI). Foram incluídas crianças de 4 a 18 anos com diagnóstico de SNRE de acordo com as diretrizes da Sociedade Indiana de Nefrologia Pediátrica, enquanto pacientes com SNRE em terapia farmacológica (estatinas) para hiperlipidemia no momento da inclusão foram excluídos do estudo^3^. Detalhes demográficos e clínicos, incluindo medidas antropométricas, pressão arterial e características de toxicidade por esteroides, foram registrados. Foram coletadas amostras de sangue para hemograma completo, eletrólitos séricos, ureia, creatinina, proteína total, albumina, colesterol total, colesterol de lipoproteína de baixa densidade (LDL-C), triglicerídeos, colesterol de lipoproteína de alta densidade (HDL-C), não-HDL-C e marcadores de disfunção endotelial (PCSK9, sE-selectina). Também foram coletadas amostras de urina isolada para determinação da relação proteína/creatinina urinária dos pacientes incluídos no estudo. A amostragem foi realizada no momento da inclusão e após 12 semanas de tratamento padrão. O plasma foi obtido por centrifugação da amostra sanguínea a 2800 g por 10 minutos para medição dos marcadores endoteliais. A amostra foi armazenada a -20 °C até o momento da análise. A avaliação quantitativa dos marcadores endoteliais foi realizada com a técnica ELISA, utilizando kits específicos para cada marcador (kit para PCSK9 humana, fabricado pela *Biovendor-Laboratorni Medicine A.S.*, República Tcheca; e kit para sE-selectina, fabricado pela *Shanghai Coon Koon Biotech Co. Ltd*). A medição foi realizada em equipamento Erba Lisa Scan EM para leitura de ELISA.

As crianças com SNRE receberam terapia padrão com inibidores da calcineurina (tacrolimo ou ciclosporina) e esquema de redução gradual da dose de prednisolona^3^. Todos os pacientes foram orientados a fazer modificações no estilo de vida para o manejo da dislipidemia, incluindo restrições alimentares e um regime padrão de exercícios físicos. As recomendações dietéticas consistiram na redução da ingestão de gorduras saturadas e colesterol, de acordo com as diretrizes CHILD-1, que restringem a gordura saturada a < 10% do total de calorias diárias e o colesterol a 300 mg/dia. Essas restrições são ainda mais rigorosas na dieta CHILD-2, na qual a ingestão de gordura saturada deve ser < 7% e a de colesterol, < 200 mg/dia^16^. O tempo de lazer diante de telas foi limitado a menos de 2 horas por dia, sendo também recomendada a prática de atividade física moderada por 1 hora/dia e atividade física vigorosa pelo menos 3 dias por semana^16^. A dieta foi registrada duas vezes por semana utilizando o método de registro estimado de avaliação dietética em tabelas alimentares estruturadas fornecidas aos pacientes. A ingestão alimentar foi avaliada em cada consulta de seguimento para garantir a adesão ao tratamento. Além de modificações no estilo de vida, foi prescrita terapia farmacológica com atorvastatina para crianças com colesterol LDL sérico > 160 mg/dl no momento da inclusão^15,16,17^. O medicamento foi administrado a crianças com 4 anos ou mais, na dose fixa de 10 mg/dia^11,15^. Estatura, peso, CPK-MB sérico, SGOT sérico e SGPT foram monitorados a cada 4 semanas em pacientes que receberam atorvastatina. Todos receberam terapia com enalapril (0,2-0,3 mg/kg) por seu efeito antiproteinúrico. Anti-hipertensivos foram prescritos para o manejo da hipertensão^3^.

A medida do desfecho primário foi a alteração nos níveis plasmáticos de colesterol total, LDL-C, E-selectina e PCSK9 em crianças com SNRE após 12 semanas de tratamento padrão. Já a medida do desfecho secundário foi a correlação dos níveis plasmáticos de E-selectina e PCSK9 com colesterol total, LDL-C, não-HDL-C, IMC, PA, TFGe, relação proteína/creatinina urinária em amostra isolada e albumina sérica no diagnóstico de SNRE e após 12 semanas de tratamento padrão.

O tamanho da amostra foi calculado por meio do software Epi Info. Entre os pacientes com síndrome nefrótica, o nível médio de PCSK9 no plasma foi de 15,13, com desvio padrão de 4,99, valor significativamente superior ao observado em controles pareados por idade e sexo (p < 0,001) em um estudo prévio realizado por Jin et al.^18^. Considerando uma diferença média de 5,67, tal como relatado neste estudo, obteve-se uma amostra de 12 pacientes para PCSK9 com poder de 80% e intervalo de confiança de 95%. Utilizando os valores de referência para a média e o DP da E-selectina de um estudo anterior realizado em nossa instituição, o tamanho da amostra foi estimado em 10. No presente estudo, recrutamos uma amostra maior composta por 34 pacientes com SNRE.

### Análise Estatística

Os dados foram analisados utilizando o *IBM SPSS Statistics* (versão 23.0). A significância estatística foi considerada para p < 0,05. Os dados categóricos foram apresentados como frequências e porcentagens. Os dados contínuos foram apresentados como média e desvio padrão, para dados com distribuição normal, ou mediana e intervalo interquartil, para distribuição não normal. O teste de *Kolmogorov-Smirnov* foi utilizado para verificar a normalidade das variáveis quantitativas. Para comparação dos pacientes nos dois momentos de avaliação, utilizou-se o teste t pareado para dados com distribuição normal e o teste de *Wilcoxon* pareado para dados com distribuição assimétrica. A correlação linear entre duas variáveis contínuas foi avaliada por meio da correlação de *Pearson* (para dados com distribuição normal) e da correlação de *Spearman* (para dados com distribuição não normal). Equações de estimação generalizadas foram empregadas para comparação entre dois grupos ao longo do tempo.

## Resultados

O grupo de estudo foi composto por 34 pacientes com diagnóstico de SNRE, acompanhados prospectivamente por 12 semanas. A idade média ao início da síndrome nefrótica foi de 63 meses, enquanto a idade média no momento do diagnóstico de SNRE foi de 88 meses. Alterações no perfil lipídico foram identificadas em 24 (71%) pacientes no momento do diagnóstico de SNRE. Nessa ocasião, 14 (41%) apresentavam hipertensão, dos quais 13 (38%) foram classificados como estágio I e 1 paciente (3%) foi classificado como estágio II. Observou-se uma melhora significativa nos percentis de PA ao longo das 12 semanas. A PA normalizou-se em 12 pacientes com hipertensão estágio I e no único paciente com hipertensão estágio II (p = 0,004) após 12 semanas de intervenção. Adicionalmente, foi realizada uma comparação entre as alterações nos níveis de E-selectina e PCSK9 entre os subgrupos dos percentis de PA (50–90%, 90–95%, 95–95+12% e > 95+12%) utilizando o teste de Kruskal Wallis, não tendo sido observada qualquer diferença significativa (p = 0,239 e p = 0,913) nos quatro subgrupos. Ao avaliar a ingestão alimentar durante o período de 12 semanas do estudo, a ingestão média diária de gordura foi de 24,93 g/dia, representando 18,9% da ingestão calórica total. A ingestão média diária de gordura saturada foi de 7,6 g/dia, correspondendo a 6,3% da ingestão calórica total. A ingestão média diária de colesterol foi de 55,42 mg, estando todos os valores dentro da faixa recomendada (Tabela 1). Houve uma melhora acentuada nos parâmetros antropométricos (Tabela 2). A relação proteína-creatinina urinária em amostra isolada apresentou redução significativa (p < 0,001), observando-se melhora nos níveis séricos de albumina (p = 0,002), conforme demonstrado na Tabela 2. A remissão completa foi alcançada em 30% dos indivíduos, enquanto 56% dos pacientes alcançaram remissão parcial. Verificou-se uma redução significativa nos níveis médios de colesterol total, LDL-C, triglicerídeos e colesterol não-HDL após 12 semanas de modificações na dieta e no estilo de vida associadas à terapia com atorvastatina (Tabela 3). Tanto a PCSK9 quanto a E-selectina demonstraram uma redução significativa (p < 0,008 e p < 0,029 para PCSK9 e E-selectina, respectivamente). As crianças que receberam atorvastatina adicionalmente às modificações no estilo de vida apresentaram maior redução no colesterol sérico, colesterol LDL, triglicerídeos e colesterol não-HDL em comparação com aquelas que realizaram somente modificações no estilo de vida. Os níveis séricos dos marcadores endoteliais (PCSK9 e E-selectina) exibiram tendência de declínio (não estatisticamente significativa) no grupo submetido à terapia com estatina (Tabela 4).

**Tabela 1:** Ingestão alimentar durante as 12 semanas de alterações no estilo de vida

Dieta	Média (DP)	Mediana (IIQ)
Ingestão Calórica Média Diária (Kcal)	1193,85 ± 323,42	1181,00 (1000,00–1320,00)
Ingestão média diária de proteínas (g)	43,41 ± 10,33	41,00 (39,00–47,50)
Déficit calórico (%)	31,07 ± 14,04	27,00 (21,00–43,00)
Déficit proteico (%)	8,22 ± 12,04	0,00 (0,00–12,50)
Ingestão média diária de gorduras (g)	24,93 ± 9,59	23,00 (16,50–33,00)
Calorias provenientes de gorduras (%)	18,90 ± 6,15	17,00 (14,50–24,35)
Ingestão média diária de gorduras saturadas (g)	7,60 (5,50–10,50)	7,60 (5,50–10,50)
Calorias provenientes de gorduras saturadas (%)	6,30 ± 3,05	6,00 (4,05–7,50)
Ingestão média diária de colesterol (mg)	55,42 ± 50,92	49,00 (20,00–60,00)

**Tabela 2:** Parâmetros antropométricos e laboratoriais no momento do diagnóstico e após 12 semanas

S. No.	Parâmetro	Casos de SNRE no momento do diagnóstico (n = 34)	Casos de SNRE em 12 semanas (n = 34)	Valor de p
1.	Peso (kg)	23,35 ± 8,25 22,10 (16,60–26,60)	22,01 ± 8,25 20,30 (16,00–25,10)	0,003^1^
2.	Altura (cm)	115,32 ± 18,63 113,90 (100,50–131,20)	117,53 ± 18,62 115,00 (102,00–132,80)	<0,001^2^
3.	IMC (kg/m^2^)	17,10 ± 2,04 16,90 (15,70–18,00)	15,39 ± 1,74 15,50 (14,00–16,40)	<0,001^1^
4.	Ureia (mg/dL)	35,41 (26,16) 30,00 (21,18–44,28)	25,60 (19,35) 21,50 (17–26)	<0,010^1^
5.	Creatinina (mg/dL)	0,36 (0,19) 0,30 (0,21–0,45)	0,34 (0,11) 0,30 (0,28–0,40)	<0,820^1^
6.	TFGe (mL/min/1,75 m^2^)	154,71 (58,34) 146,00 (123–196)	151,91 (45,19) 144,00 (127–169)	<0,775^1^
7.	Proteína Total (g/dL)	5,47 (1,26) 5,60 (4,26–6,47)	6,47 (1,08) 6,80 (6,40–7,20)	<0,001^1^
8.	Albumina (g/dL)	3,01 (1,06) 3,10 (2,10–3,88)	3,67 (1,03) 4,00 (3,80–4,26)	<0,002^1^
9.	uP:uC	4,77 (6,47) 2,42 (1,72–7,22)	1,17 (2,06) 0,48 (0,19–0,98)	<0,001^1^

Notas – Os valores são apresentados como média ± DP e mediana ± IIQ. *Considerou-se significância estatística para p < 0,05.

^1^Teste de *Wilcoxon* pareado;

^2^Teste t pareado.

**Tabela 3:** Perfil lipídico, níveis de e-selectina e pcsk9 no momento do diagnóstico e após 12 semanas

S. No.	Parâmetro	Casos de SNRE no momento do diagnóstico	Casos de SNRE em 12 semanas	Valor de p
1.	Colesterol total (mg/dL)	273,71± 124,70 225,50 (188,00–312,00)	177,59 ± 91,66 159,50 (131,25–180,75)	<0,001^1^
2.	LDL (mg/dL)	160,50 ± 91,88 126,50 (95,50–213,00)	100,85 ± 75,74 75,00 (63,50–112,75)	<0,001^1^
3.	Colesterol não-HDL (mg/dL)	193,00 ± 119,96 145,50 (112,25–246,00)	118,03 ± 88,39 94,00 (77,50–118,75)	<0,001^1^
4.	TG (mg/dL)	234,38 ± 138,13 214,50 (120,00–335,00)	150,60 ± 106,13 127,00 (87,50–172,25)	0,001^1^
5.	HDL (mg/dL)	80,71± 22,82 81,00 (61,50–97,25)	59,56 ± 11,82 58,00 (541,50–68,00)	<0,001^2^
6.	E-selectina (pg/ml)	614,24 ± 112,38 631,25 (575,25–668,00)	544,37 ± 137,91 561,50 (427,50–657,75)	0,029^1^
7.	PCSK9 (ng/mL)	518,25 ± 222,34 521,90 (388,25–706,35)	373,25 ± 198,71 326,50 (217,25–504,25)	0,008^1^

Notas – Os valores são apresentados como média ± DP e mediana ± IIQ. *Considerou-se significância estatística para p < 0,05.

^1^Teste de *Wilcoxon* pareado;

^2^Teste t pareado.

**Tabela 4:** Análise de subgrupos de pa cientes com e sem tratamento com estatina no momento basal e após 12 semanas

S. No.	Parâmetro	Estatina	Basal	12 semanas	Valor de p	Valor p total^3^
1.	Colesterol (mg/dL)	Sim (n = 16)	354,56 (136,61)	201,38 (121,01)	0,002^1^	
		Não (n = 18)	201,83 (45,80)	156,44 (48,82)	0,001^1^	0,006
2.	Colesterol LDL (mg/dL)	Sim (n = 16)	220,12 (99,40)	125,88 (99,66)	0,012^1^	
		Não (n = 18)	107,50 (36,73)	78,61 (35,46)	0,001^1^	0,021
3.	Colesterol não-HDL (mg/dL)	Sim (n = 16)	267,62 (135,53)	143,69 (117,92)	0,006^1^	
		Não (n = 18)	126,67 (42,76)	95,22 (41,54)	0,004^1^	0,019
4.	Triglicerídeos (mg/dL)	Sim (n = 16)	311,00 (146,99)	199,12 (131,54)	0,013^1^	
		Não (n = 18)	166,28 (86,81)	107,39 (49,04)	0,053^1^	0,226
5.	Colesterol HDL (mg/dL)	Sim (n = 16)	86,94 (25,58)	75,17 (19,10)	<0,001^2^	
		Não (n = 18)	57,69 (11,43)	61,22 (12,23)	0,012^2^	0,040
6.	Albumina (g/dL)	Sim (n = 16)	2,69 (1,04)	3,37 (1,24)	0,083^1^	
		Não (n = 18)	3,28 (1,03)	3,94 (0,73)	<0,001^1^	0,977
7.	Relação proteína/creatinina urinária	Sim (n = 16)	6,72 (8,76)	1,62 (2,71)	<0,001^1^	
		Não (n = 18)	3,04 (2,61)	0,76 (1,18)	<0,001^1^	0,089
8.	PCSK9 (ng/mL)	Sim (n = 16)	509,11 (237,74)	425,19 (198,10)	0,252^1^	
		Não (n = 18)	526,38 (214,34)	327,08 (192,94)	0,021^1^	0,251
9.	E-selectina (pg/mL)	Sim (n = 16)	583,03 (111,71)	557,34 (130,67)	0,528^1^	
		Não (n = 18)	641,97 (108,50)	532,83 (146,81)	0,021^1^	0,151

Notas – Os valores são apresentados como média ± DP. *Considerou-se significância estatística para p < 0,05.

^1^Teste de *Wilcoxon* pareado;

^2^Teste t pareado;

^3^Equação de estimação generalizada.

A variação percentual nos parâmetros do perfil lipídico e nos marcadores de disfunção endotelial foi comparada com o estado de remissão, tendo-se observado uma variação semelhante para o colesterol total (p = 0,180), LDL-C (p = 0,257), triglicerídeos (p = 0,355), não-HDL-C (p = 0,097), PCSK9 (p = 0,138) e E-selectina (p = 0,902) nos subgrupos sem remissão, com remissão parcial e com remissão completa (Tabela 5). A PCSK9 apresentou correlação positiva significativa com a relação proteína/creatinina urinária (r = 0,36; p = 0,035) e correlação negativa significativa com a albumina sérica (r = –0,33; p = 0,054) às 12 semanas. A E-selectina apresentou uma correlação positiva com o IMC no momento do diagnóstico de SNRE (r = 0,52; p = 0,002). Em pacientes que receberam atorvastatina, não foi observada elevação significativa nos níveis séricos de creatinina quinase e transaminases durante o acompanhamento de 12 semanas. Foi observada uma melhora significativa no peso e na altura dos pacientes após 12 semanas. Não foram registrados outros eventos adversos no subgrupo em terapia com atorvastatina.

**Tabela 5:** Comparação do perfil lipídico, e-selectina e pcsk9 com o estado de remissão

S. No.	Parâmetro (Variação percentual)	Estado de Remissão			Valor de p
		Nenhuma (n = 5)	Parcial (n = 19)	Completa (n = 10)	
1.	LDL	--25,31 (-31,71– -15,25)	-29,56 (-52,32–8,12)	-43 (-50,82– -32,34)	0,257
2.	Colesterol Total	-24,17 (-26,12– -13,76)	-27,81 (-48,06– -9,11)	-38,32 (-53,68– -29,73)	0,180
3.	Triglicerídeos	-20,87 (-44,08– -20,42)	-27,86 (-56,17–13,09)	-56,24 (-75,47– -4,81)	0,355
4.	Colesterol não-HDL	-21,31 (-27,41– -11,78)	-22,96 (-50,42–6,67)	-46,58 (-59,22– -37,14)	0,097
5.	E-Selectina	5,56 (-31,17–6,49)	-12,09 (-23,46–9,33)	-7,16 (-21,03– -3,78)	0,902
6.	PCSK9	-24,66 (-29,25–28,17)	-29,64 (-47,95–23,46)	-60,27 (-79,36– -21,58)	0,138

Notas – Os valores são apresentados como mediana ± IIQ. *Considerou-se significância estatística para p < 0,05. Teste de *Kruskal-Wallis*.

## DISCUSSãO

A lesão endotelial na síndrome nefrótica resulta de uma variedade de causas, incluindo hipoalbuminemia, dislipidemia e estresse oxidativo^13^. As anormalidades lipídicas na síndrome nefrótica ocorrem devido ao aumento da biossíntese de lipídios e à redução da depuração, sendo esta última o fator predominante. Os níveis plasmáticos de colesterol total, LDL-C, colesterol de lipoproteína de densidade muito baixa (VLDL-C) e triglicerídeos encontram-se aumentados e permanecem persistentemente elevados em crianças com SNRE^5^. A exposição a fatores aterogênicos, incluindo o aumento dos níveis séricos de lipídios, altera o fenótipo endotelial para um estado não adaptativo conhecido como disfunção endotelial. Esse processo resulta na liberação de diversas moléculas pró-inflamatórias e pró-trombóticas a partir das células endoteliais ativadas, incluindo vWF, tPA, VCAM-1 e E-selectina^19^. A PCSK9 é uma serina protease produzida principalmente pelo fígado. Ela se liga ao domínio extracelular do complexo LDL-LDL-R nos hepatócitos, impedindo sua reciclagem. Além disso, a PCSK9 interage com os receptores *scavenger* CD36 presentes nos macrófagos para a captação da LDL oxidada e, portanto, pode desempenhar um papel direto na promoção da aterosclerose^20^.

Anomalias subclínicas nos vasos sanguíneos e no miocárdio começam a se desenvolver durante a infância e se manifestam clinicamente como infarto do miocárdio, angina e acidentes cerebrovasculares, incluindo tromboembolismo, em fases posteriores da vida^4,21^. Nosso estudo demonstrou que os marcadores de disfunção endotelial, PCSK9 e E-selectina, diminuíram significativamente após 12 semanas de modificação na dieta e no estilo de vida, indicando que a regulação dietética e a prática adequada de atividade física desempenham um papel importante na reversão da disfunção endotelial em crianças com síndrome nefrótica. Ao avaliar a composição da dieta de nossos pacientes, observou-se, no início do estudo, uma ingestão de gordura saturada significativamente superior à recomendada. No entanto, com o acompanhamento periódico do cumprimento das modificações recomendadas e aconselhamento nutricional recorrente, a maioria dos pacientes reduziu a ingestão alimentar para os níveis recomendados. Dessa forma, o envolvimento de um nutricionista e a adesão ao plano alimentar são essenciais.

Os efeitos das alterações no estilo de vida sobre o tratamento da dislipidemia e da disfunção endotelial variam. Woo et al.^22^ investigaram o impacto de uma dieta hipocalórica, com baixo teor de gordura, associada à prática de exercícios físicos, sobre a vasodilatação mediada pelo fluxo e relataram uma redução do colesterol sérico, acompanhada de melhora da função endotelial no curto prazo (6 semanas), com reversão para níveis quase normais após intervenção sustentada ao longo de um ano. Engler et al.^23^ avaliaram os efeitos da dieta *Step II* do *National Cholesterol Education Program* (Programa Nacional de Educação sobre Colesterol) na vasodilatação dependente do endotélio em crianças com dislipidemia. As modificações na dieta reduziram significativamente a concentração de LDL-C, mas não promoveram melhora na função endotelial durante o estudo de 6 meses. A análise dos subgrupos de pacientes segundo o estado de remissão — sem remissão (14%), remissão parcial (30%) e remissão completa (56%) — não revelou diferença significativa na variação dos níveis de colesterol total, colesterol LDL, triglicerídeos e colesterol não-HDL após 12 semanas de tratamento padrão, sugerindo que o estado de remissão não exerce impacto significativo na melhora da disfunção endotelial.

É amplamente reconhecido que a dislipidemia pode alterar o fenótipo endotelial, resultando em disfunção endotelial. Em nosso estudo, 71% dos pacientes apresentavam perfil lipídico alterado no início da SNRE, com aumento do colesterol total em 71% dos pacientes e colesterol LDL elevado em 47% deles. Watts et al.^24^ demonstraram que a disfunção endotelial em pacientes com síndrome nefrótica é semelhante àquela observada em indivíduos com hiperlipidemias, demonstrando uma correlação significativamente positiva com o LDL sérico, o que levou à conclusão de que a dislipidemia desempenha papel relevante na disfunção endotelial. Em nosso estudo, a média do colesterol total (177,59 mg/dL) e do colesterol LDL (100,85 mg/dL) após o tratamento foi semelhante à dos controles no estudo de Watts et al.^19^. Diversos trabalhos demonstraram que pacientes com síndrome nefrótica apresentam níveis séricos de PCSK9 superiores aos dos controles, havendo correlação positiva com o colesterol total e o colesterol HDL no momento basal, indicando que a PCSK9 desempenha um papel significativo na contribuição para a dislipidemia^18,25^. Observou-se, ainda, uma correlação positiva entre os níveis séricos de PCSK9 e a relação proteína/creatinina urinária e uma correlação negativa com a albumina sérica após o tratamento. Doiron et al.^26^ encontraram uma correlação semelhante entre a proteinúria e os níveis de PCSK9 em pacientes com proteinúria em faixa nefrótica e não nefrótica, bem como em controles. O presente estudo não identificou uma correlação significativa entre os marcadores endoteliais e os níveis de colesterol total e colesterol LDL.

Crianças com síndrome nefrótica também enfrentam infecções recorrentes, estresse oxidativo, hipertensão e toxicidade medicamentosa associada tanto aos corticosteroides quanto aos imunossupressores poupadores de esteroides, como os inibidores da calcineurina, fatores que aumentam o risco de disfunção endotelial e eventos cardio-vasculares^27,28^. Em nosso estudo, 41% dos pacientes apresentavam hipertensão no momento do diagnóstico de SNRE, com uma melhora significativa no percentil da PA ao longo de 12 semanas. Sabe-se que a hipertensão, por diversos mecanismos, pode causar disfunção endotelial^29^. Não observamos diferença estatisticamente significativa na variação dos níveis de E-selectina e PCSK9 entre pacientes com e sem hipertensão arterial.

Em nosso estudo, foi prescrito tacrolimo a todos os pacientes com SNRE. Pełkowska et al.^30^ compararam a disfunção endotelial em pacientes com SNRE em remissão, sob terapia com ciclosporina, e em pacientes nefróticos com recidiva, relatando disfunção endotelial semelhante, o que indica que os ICN desempenham papel importante na disfunção endotelial. Estudos realizados em pacientes pós-transplante renal, avaliando o efeito da ciclosporina e do tacrolimo sobre a função endotelial, relataram que o tacrolimo causa menos disfunção endotelial em comparação à ciclosporina^31,32^. Todos os pacientes em nosso estudo receberam inibidores da ECA devido ao seu efeito antiproteinúrico. Tais inibidores, ao aumentar a disponibilidade de óxido nítrico e reduzir o estresse oxidativo e a inflamação mediada pela LDL oxidada, desempenham um papel significativo na melhora da disfunção endotelial^29^. Não realizamos análises genéticas em nossos pacientes; portanto, não foi possível avaliar se a origem genética ou imunológica da doença exerce impacto nas crianças com SNRE. Kaukinen et al.^33^ avaliaram o endotélio glomerular em crianças com síndrome nefrótica congênita (mutação NPHS-1) e demonstraram que a expressão de moléculas de adesão, como a molécula de adesão intercelular 1 (ICAM-1), estava aumentada nos glomérulos renais NPHS1-positivos em comparação com os controles. Em crianças com GESF, as células endoteliais circulantes e o fator de crescimento transformador beta estão significativamente elevados^34^.

Foi realizada uma análise de subgrupos entre crianças que receberam atorvastatina e aquelas que não a utilizaram, além do manejo dietético recomendado a todas como parte do tratamento padrão para dislipidemia. A atorvastatina foi administrada a uma dose fixa de 10 mg. Não foram observados efeitos adversos clínicos ou bioquímicos em nenhum paciente em terapia com atorvastatina. Múltiplos estudos demonstraram a segurança das estatinas em pacientes pediátricos com hipercolesterolemia^35,36^. A tendência de alteração nos marcadores de disfunção endotelial, no perfil lipídico, na relação proteína-creatinina urinária e na albumina ao longo de 12 semanas de terapia foi comparada entre os dois grupos. Observou-se uma redução significativa no colesterol total, colesterol LDL e colesterol não-HDL tanto em pacientes que receberam estatinas quanto naqueles que não receberam. No entanto, verificou-se uma diferença significativa na tendência de redução desses parâmetros entre os dois grupos, sugerindo que as estatinas de fato desempenham papel importante no tratamento da dislipidemia. Houve uma diminuição significativa da proteinúria (relação proteína/creatinina urinária em amostra isolada) nos subgrupos em uso de estatina e sem estatina; no entanto, a redução da proteinúria foi semelhante em ambos os subgrupos. Coleman e Watson^37^ relataram um efeito significativo e sustentado da sinvastatina, enquanto a intervenção alimentar teve apenas um efeito modesto no perfil lipídico. Os marcadores de disfunção endotelial apresentaram tendência à redução; porém, essa redução não alcançou significância estatística no grupo de pacientes que receberam estatinas ao longo de 12 semanas de terapia, sugerindo que a disfunção endotelial persiste, ao menos no curto prazo, em pacientes com SNRE, apesar desse tratamento. Deve-se avaliar se a extensão da terapia para além de 12 semanas em pacientes com SNRE contribui para a redução da disfunção endotelial.

## CONCLUSãO

Modificações na dieta e no estilo de vida levaram a uma redução nos marcadores de disfunção endotelial e a uma melhora nos parâmetros do perfil lipídico em pacientes com SNRE. Modificações no estilo de vida, incluindo restrições alimentares e aumento da atividade física, constituem o primeiro passo na prevenção e no tratamento da dislipidemia e de outros fatores de risco para doenças cardiovasculares. No entanto, a adesão a essas mudanças representa um desafio e requer monitoramento periódico. O uso da terapia com estatinas contribuiu para a melhora do perfil lipídico; contudo, no curto prazo, as estatinas não reduziram significativamente os níveis dos marcadores de disfunção endotelial. Nosso estudo foi limitado pelo curto período de acompanhamento. São necessários estudos prospectivos de longo prazo sobre os efeitos das estatinas na disfunção endotelial, bem como sobre o uso de novas terapias, como os inibidores de PCSK9, uma vez que esses medicamentos podem atuar como importantes alvos para atenuar a disfunção endotelial em crianças com proteinúria.

## Data Availability

Os conjuntos de dados gerados e analisados durante o presente estudo estão disponíveis mediante solicitação ao autor correspondente.
